# Comparison Of Transfer Accuracy Among Hexed Implant Mounts, Clips Impression Copings, And Open-Tray Impression Copings at Different Implant Angulations: An In Vitro Study

**DOI:** 10.1186/s12903-025-06304-8

**Published:** 2025-06-06

**Authors:** Merna M. Seif, Ahmed A. Abdel Hakim, Hassan M. Abouelkheir, Rana A. Negm

**Affiliations:** 1 Department of Prosthodontics, Faculty of Dentistry, Alexandria University, Champollion St., Azarita, Alexandria, 21527 Egypt; 2 Department of Oral Medicine, Periodontology, and Radiology, Faculty of Dentistry, Alexandria University, Alexandria, Egypt

**Keywords:** Dental implants, Impression accuracy, Open-tray, Clips impression coping, Hexed implant mount, Snap-on technique

## Abstract

**Background:**

Accurate impression registration is essential for transferring the three-dimensional (3D) implant position to the definitive cast, ensuring passivity of the final prosthesis. Various impression techniques have been developed to optimize accuracy, particularly for angulated implants. This study aimed to evaluate and compare the accuracy of open-tray impression copings, clips impression copings, and hexed implant mounts in transferring implant positions for both straight and angulated implants.

**Methods:**

Five implants with different angulations (three at 0°, one at 15°, and one at 25°) were placed in an epoxy resin model, reflecting angulations commonly encountered in clinical practice. Thirty impressions were made using three types of impression copings: open-tray, clips (closed-tray), and hexed implant mounts (closed-tray), with ten impressions per group. Impressions were poured, and CBCT scans of the reference model and casts were obtained. The resulting DICOM files were converted to STL format using reverse engineering software to evaluate implant position accuracy based on shoulder deviation, apical deviation, angular deviation, and vertical shift. A significance level of *p* < 0.05 was set. One-way ANOVA and post hoc tests were performed; Tukey’s HSD was applied when variance homogeneity was met, while the Games-Howell test was used when this assumption was violated.

**Results:**

The study revealed that among the three coping types, the hexed implant mount demonstrated significantly higher angular deviation (*p* < 0.001), apical deviation (*p* = 0.003), and vertical shift (*p* < 0.001) for 25° angulated implants compared to the open-tray and clips copings. There were no significant differences between the open-tray and clips groups at this angulation. At 15° angulation, the hexed implant mount showed a significantly greater vertical shift (*p* = 0.011) compared to the open-tray coping, while no significant difference was observed between the clips and open-tray copings. For straight implants (0° angulation), all three coping types—open-tray, clips, and hexed implant mounts—showed no significant differences in any measured parameter.

**Conclusions:**

Open-tray and clips impression copings provide reliable implant position transfer for straight and angulated implants up to 25°. The hexed implant mount is accurate up to 15° angulation but shows increased deviations at 25°.

## Background

The accuracy of impression registration is crucial in implant prosthetic treatment, directly affecting the master cast precision and passive fit of the prosthesis [1, 2]. Several factors influence accuracy, including the impression technique (direct or indirect), implant angulation, coping geometry, the type of prosthetic connection, and material properties [3–8]. While titanium remains the standard material for implant components, newer biomaterials such as polyetheretherketone (PEEK) are increasingly being introduced, with their distinct properties potentially influencing impression accuracy [9]. Any inaccuracies can compromise passive fit and long-term clinical success [10–12].

Achieving absolute passive fit remains a challenge in clinical practice [8, 13]. Unlike natural teeth, which exhibit mobility due to the periodontal ligament, dental implants are osseointegrated with minimal movement (~ 10 μm) [14]. Misfit can introduce stresses, causing complications such as screw loosening, fractures, or loss of osseointegration [15, 16]. Discrepancies as small as 50 μm can be detected in accurate impressions [17], with angular deviations of around 0.4° considered acceptable [18, 19].

Digital technology has significantly transformed prosthodontics, particularly with the use of intraoral and desktop scanners for impression registration. While these methods are effective for partial-arch impressions, full-arch scans remain challenging due to inconsistent results and difficulties in accurately merging images, especially in edentulous cases [20–26]. Consequently, conventional impression techniques often yield more predictable outcomes than digital methods in full-arch scenarios [27].

To optimize accuracy, various techniques have been developed. The open-tray technique improves accuracy by directly picking up the coping, but securing the implant analog can introduce minor inaccuracies [1, 28]. On the other hand, the closed-tray technique is beneficial in limited inter-arch space or exaggerated gag reflex; however, it is prone to misalignment [1, 28]. Both methods involve repeated screwing and unscrewing, which can lead to discomfort and potential deformation, thereby affecting accuracy [29].

The International Team for Implantology (ITI) introduced the snap-on (press-fit) technique, combining the benefits of both methods. It eliminates large tray openings and extended screws, improving efficiency and comfort; however, inaccuracies like plastic deformation may still occur [29, 30].

Hexed snap copings and hexed implant mounts, utilizing the same press-fit principle, have been developed to enhance cost-effectiveness and accuracy. Given the discrepancies in previous studies, this research aims to compare the precision of open-tray and snap-on techniques using clips impression copings and hexed implant mounts. The null hypothesis proposes no significant differences in shoulder radial deviation, apical radial deviation, angular deviation, and vertical shift among the three coping types.

## Methods

### Reference Model Fabrication

**Fig. 1:**
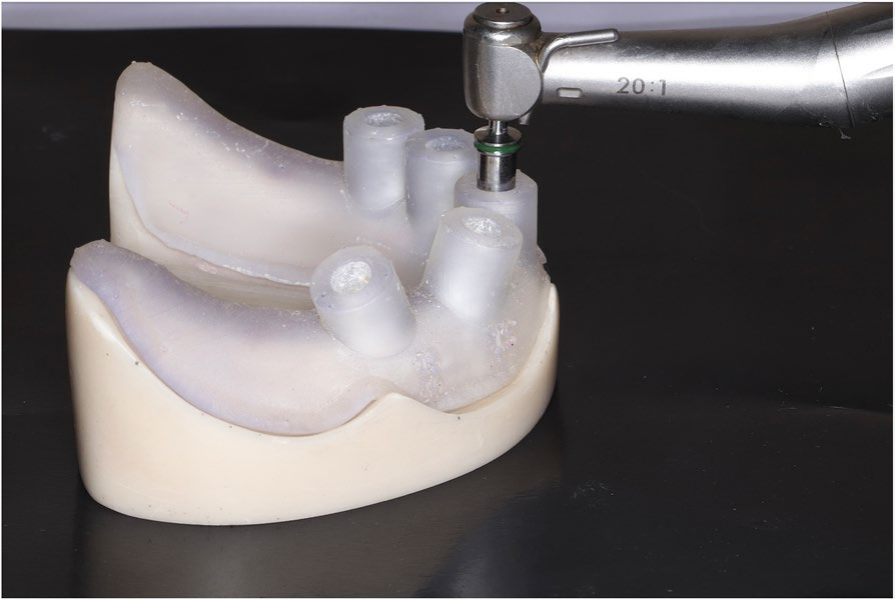
Implant drilling using 3D printed surgical guide

A mandibular edentulous epoxy resin model was scanned using a laboratory optical scanner (Medit T710, South Korea), in which the resulting STL file was imported into virtual implant planning software (Bluesky Bio, Illinois, United States) to facilitate precise placement of five implants (Biodem Implants, Düsseldorf, Germany) of equal size (diameter:3.8 mm/10 mm length). Implant positioning was planned as follows: one implant at the midline with a 0° angulation, two implants on the left side (canine and second premolar regions) also placed at 0° angulation, while on the right side, one implant was positioned in the canine region at a 15° mesial angulation, and another in the second premolar region at a 15° distal angulation with an additional 10° buccal angulation. A sleeveless surgical guide was printed using a 3D printer (Formlabs Form 4, United States) and was utilized for site marking and drilling before securing all five implants in their preplanned positions (Fig. 1), thereby establishing the reference model for the study.

### Impression Techniques and Study Groups

**Fig. 2:**
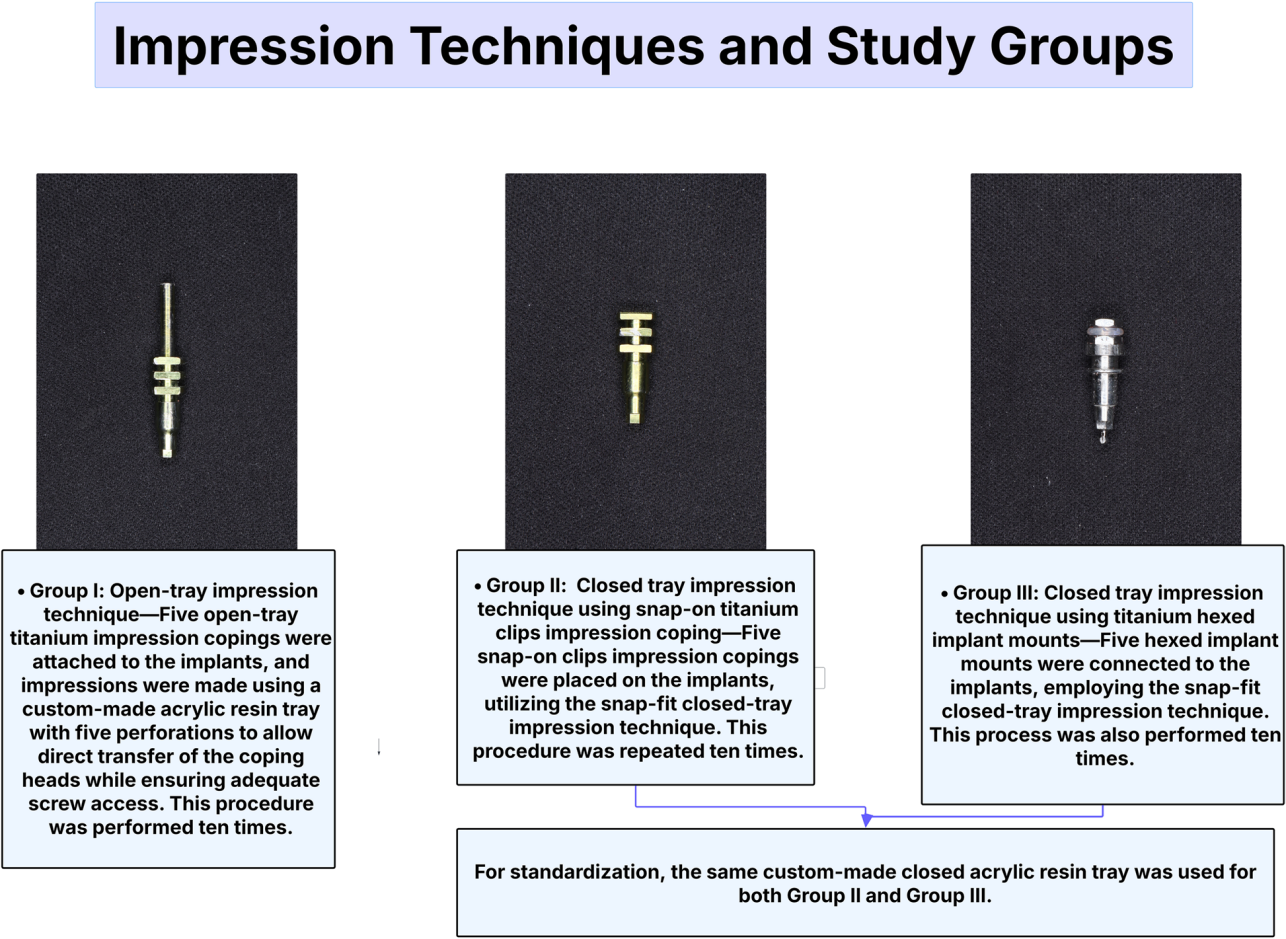
Study groups and impression techniques. Three impression techniques were evaluated: Group I used an open-tray method with open-tray titanium copings; Groups II and III employed closed-tray techniques with snap-on titanium clips and hexed implant mounts, respectively. All impressions were made ten times. A custom-made acrylic resin tray was used for Group I, while a standardized closed-tray was used for Groups II and III

All impression copings and implant mounts used in this study were made of titanium to ensure consistency in material properties across groups. Samples were divided into three groups based on the impression technique. Group I used the open-tray method with titanium open-tray copings, while Groups II and III followed the closed-tray technique using snap-on titanium clips and hexed implant mounts, respectively. Each group comprised ten impressions. For Group I, a custom acrylic resin tray with large openings to accommodate the long coping screws was utilized. On the other hand, Groups II and III used the same closed tray for standardization. An illustration of the grouping is provided in (Fig. 2).

### Sample Size Calculation

The sample size was estimated assuming a 5% alpha error (α = 0.05) and 80% study power (β = 0.20). A 95% confidence level was used to detect differences in accuracy between the open-tray and snap-on impression methods. Based on these parameters, the required sample size was determined to ensure adequate statistical power for the comparisons.

### Standardization of Impression Removal

To ensure uniformity in the force and direction of impression tray removal, a hole was incorporated into the base of the reference model to allow secure attachment to a Universal Testing Machine (UTM) (5ST, Tinius Olsen, England), which enabled controlled vertical removal of the impressions perpendicular to the occlusal plane at a constant crosshead speed of 5 mm/min. A custom metal attachment was designed and fixed to the tray to provide a stable connection to the UTM.

### Reference Model Preparation for Digital Superimposition

Three Ø2-mm metal beads were incorporated into the reference model, two of which were placed on the posterior crest of the ridge and one on the labial side, to serve as fixed reference points for digital superimposition and enable precise assessment of implant position deviations.

### Impression Material and Casts Fabrication

**Fig. 3:**
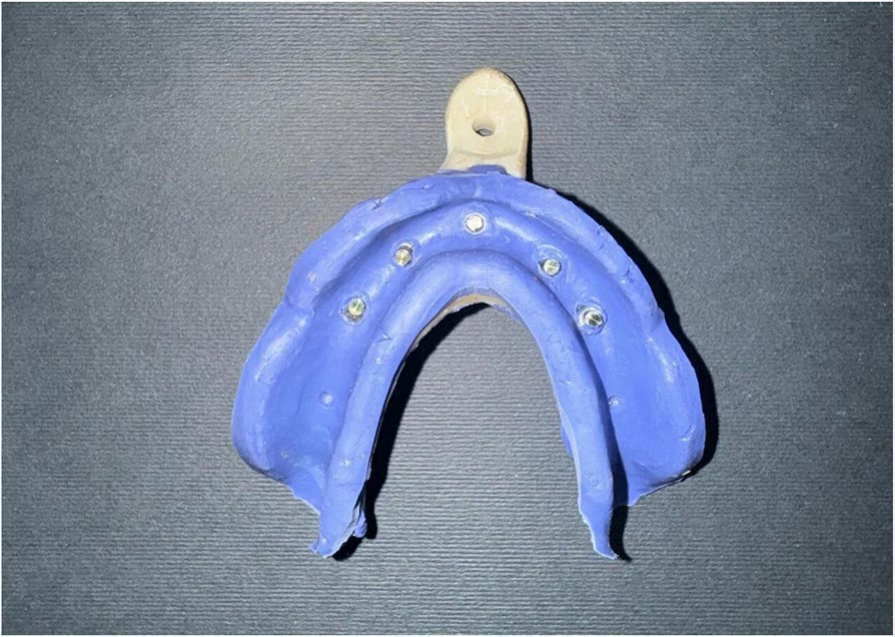
Open-tray implant impression. Impression copings are captured in VPES, showing depressions that will accommodate metal beads used for digital superimposition

All impression copings were splinted using low-shrinkage, autopolymerizing resin (Duralay Inlay Pattern Resin, Reliance Dental Manufacturing LLC, United States). To minimize polymerization shrinkage effects, the splints were sectioned after 24 h, carefully realigned, reconnected, and then left to cure for an additional 24 h before impression registration. [31–33] Vinyl polyether siloxane (VPES) medium-viscosity monophasic precision impression material (Identium Medium, Kettenbach SNC, France) was used for all impressions (Fig. 3), where a thin layer of VPES adhesive (Identium® Adhesive, Kettenbach GmbH & Co. KG., Germany) was applied to the impression tray and allowed to dry for five minutes. Subsequently, the impression material was dispensed from the Automixer Cartridge System (Kettenbach GmbH & Co. KG, Eschenburg, Germany) into the custom tray and around the transfer copings, after which the tray was positioned onto the reference model. The impression was removed vertically as previously described using the UTM at a constant crosshead speed of 5 mm/min.

Following impression procedures, all impressions were poured using extra-hard Type IV dental stone (Zermakh Elite Rock, Italy). CBCT scans of the reference model and all casts were acquired using an imaging system (Vatech Green X, Gyeonggi-do, South Korea) equipped with a metal artifact reduction (MAR) feature, with a field of view (FOV) of 8 × 5 cm and a voxel size of 0.2 mm. The obtained DICOM files were subsequently converted into STL format using 3D visualization software (3D Slicer, Massachusetts, United States). For standardization, all steps were performed by the same operator to minimize inter-operator variability.

### Implant Position Accuracy Assessment

Metal bead locations served as fixed reference points during alignment. Each of the three metal beads was initially secured to the reference model using cyanoacrylate (CYACEM, Bredent Medical GmbH & Co. KG, Senden, Germany) and verified for stability. During cast fabrication, beads of identical size were inserted into the corresponding negative spaces within the impression and fixed with cyanoacrylate. Gentle vibration was applied to ensure they remained stationary. After the cast had set, the stability of the beads was rechecked, and they were manually repositioned into the corresponding indentations of the poured cast and re-secured with cyanoacrylate. This approach enabled consistent and precise reference points for accurate measurement of implant deviations.

**Fig. 4:**
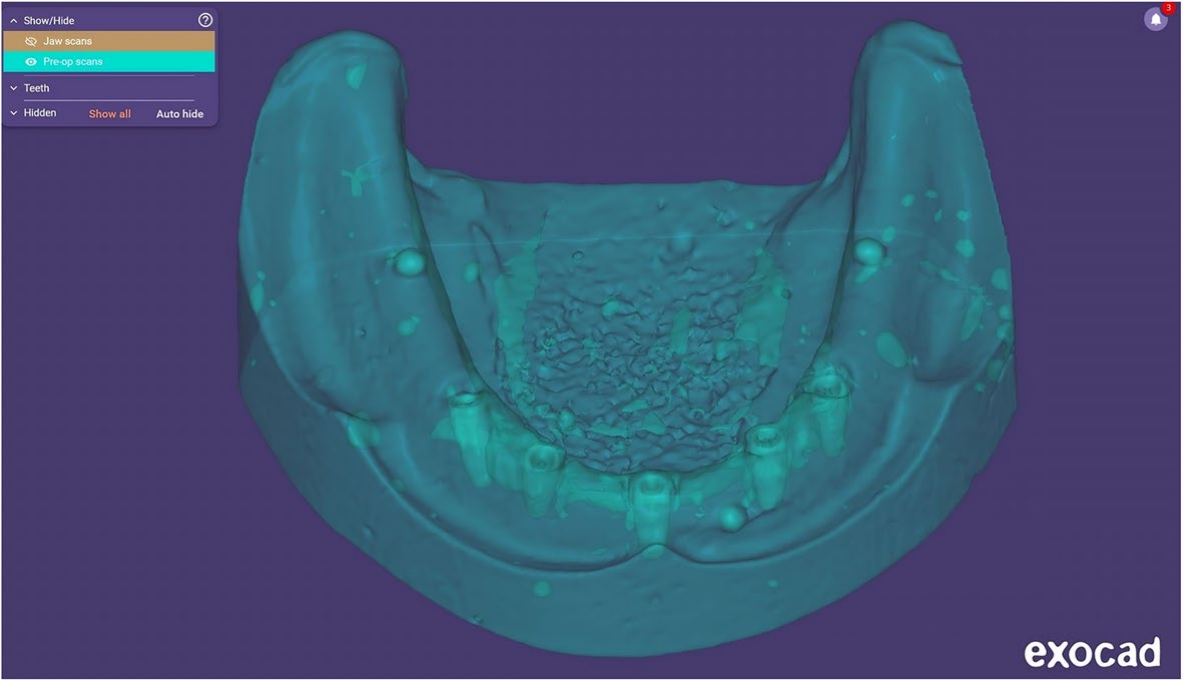
Relation between the cast STL File and the implants and metal beads STL file

**Fig. 5:**
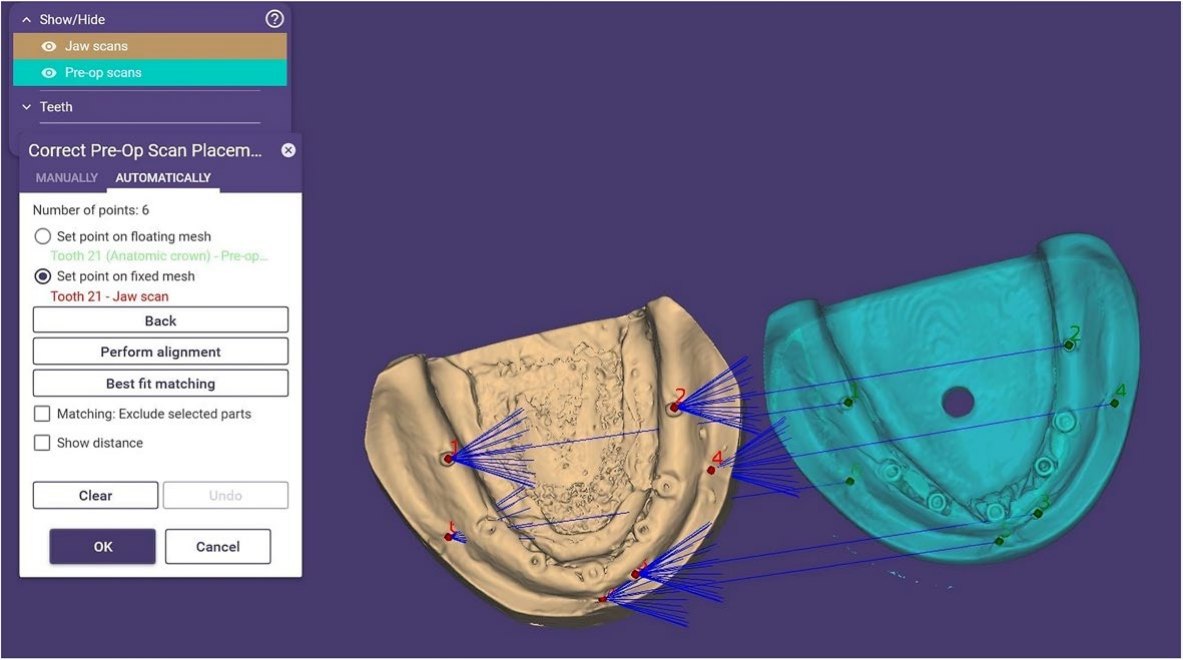
Alignment of the reference model and stone cast using multiple landmarks and metal beads

**Fig. 6:**
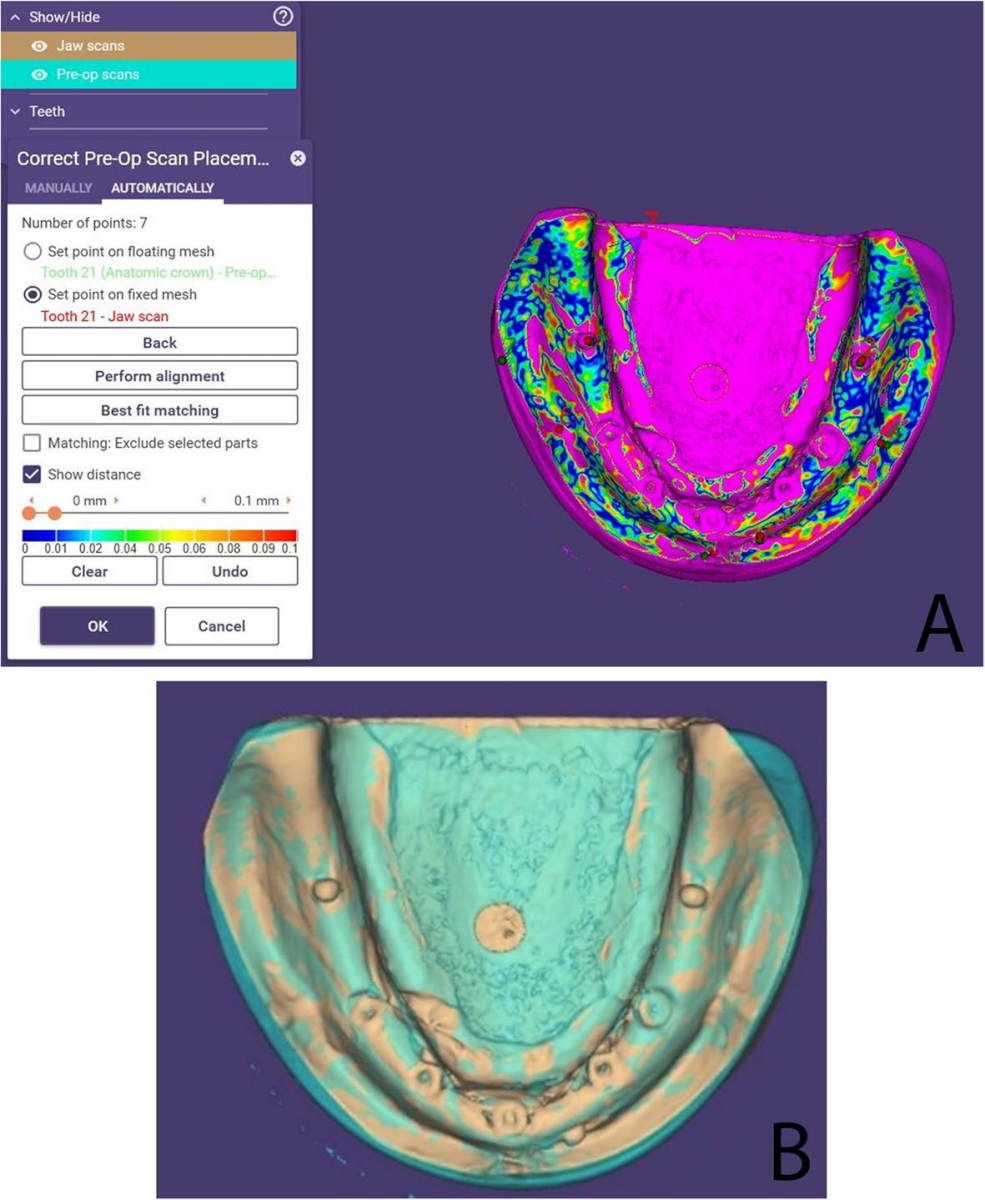
(**A**) Best-fit matching; (**B**) Alignment based on common landmarks

**Fig. 7:**
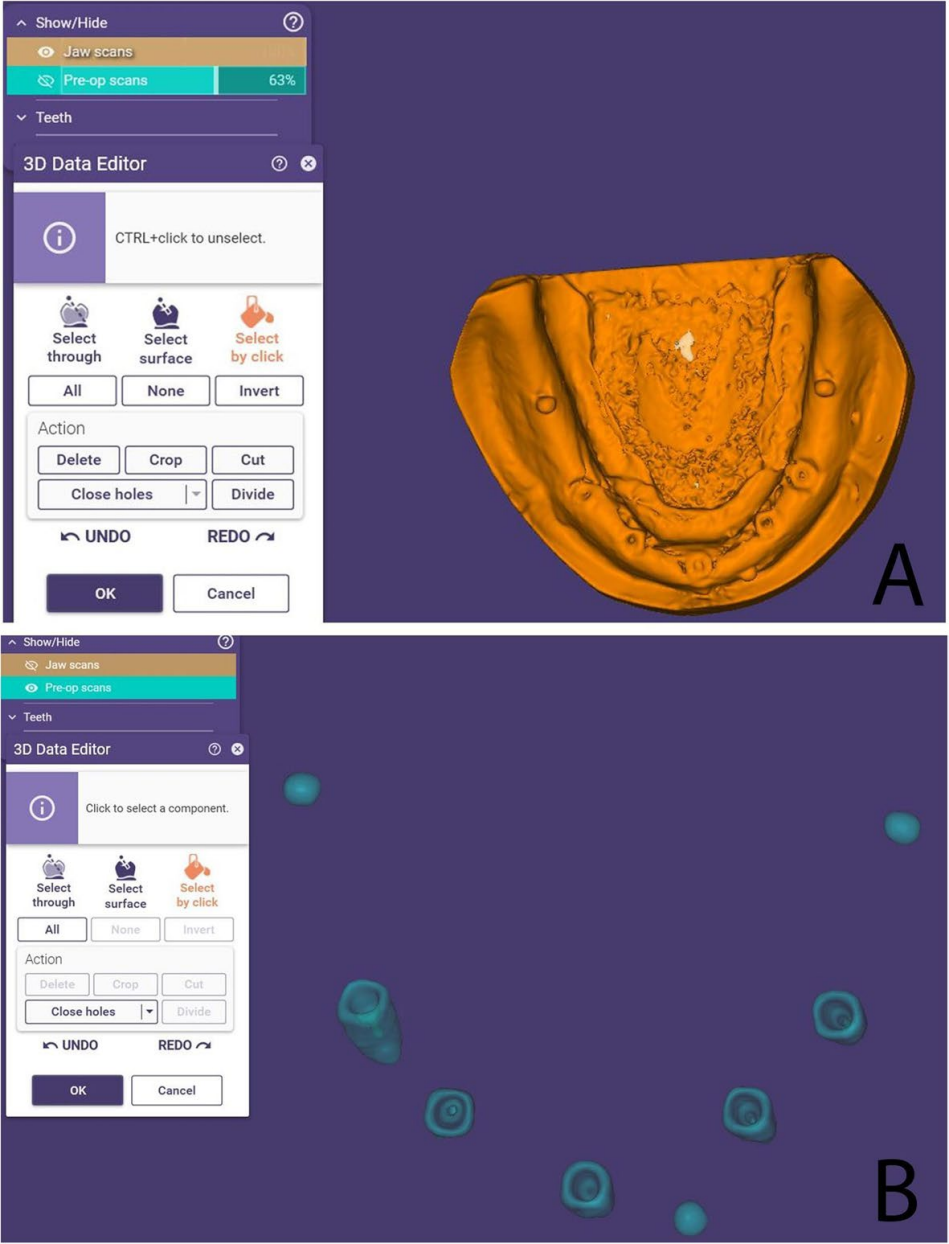
Removal of resin and stone models after alignment, leaving implants and metal beads

For digital analysis, two STL files were generated for each cast and the reference model: one representing the complete model (resin or stone) and another containing only the five implants and three metal beads (Fig. 4). Alignment was performed using ExoCAD DentalCAD 2016 (exocad GmbH, Darmstadt, Germany) by importing these STL files. Initial multi-point alignment utilized shared anatomical landmarks (e.g., arch form, ridge contour, and freni) to ensure stable superimposition between casts and the reference model (Fig. 5). Once alignment was confirmed via best-fit matching (Fig. 6A, B), non-essential scan data (resin or stone model) were digitally removed, isolating the five implants and metal beads (Fig. 7A, B).

**Fig. 8:**
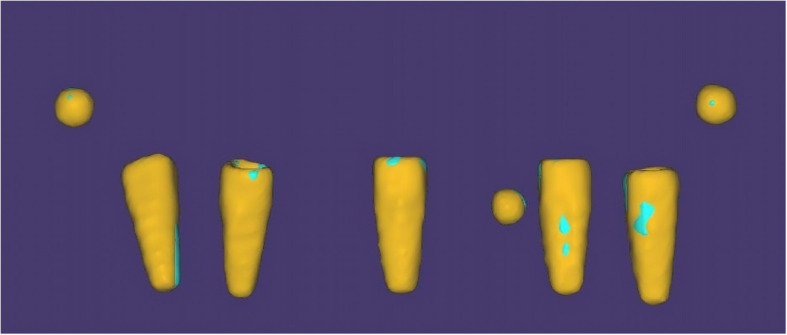
Aligned implants and metal beads STL files ready for import to Geomagic Control X

**Fig. 9:**
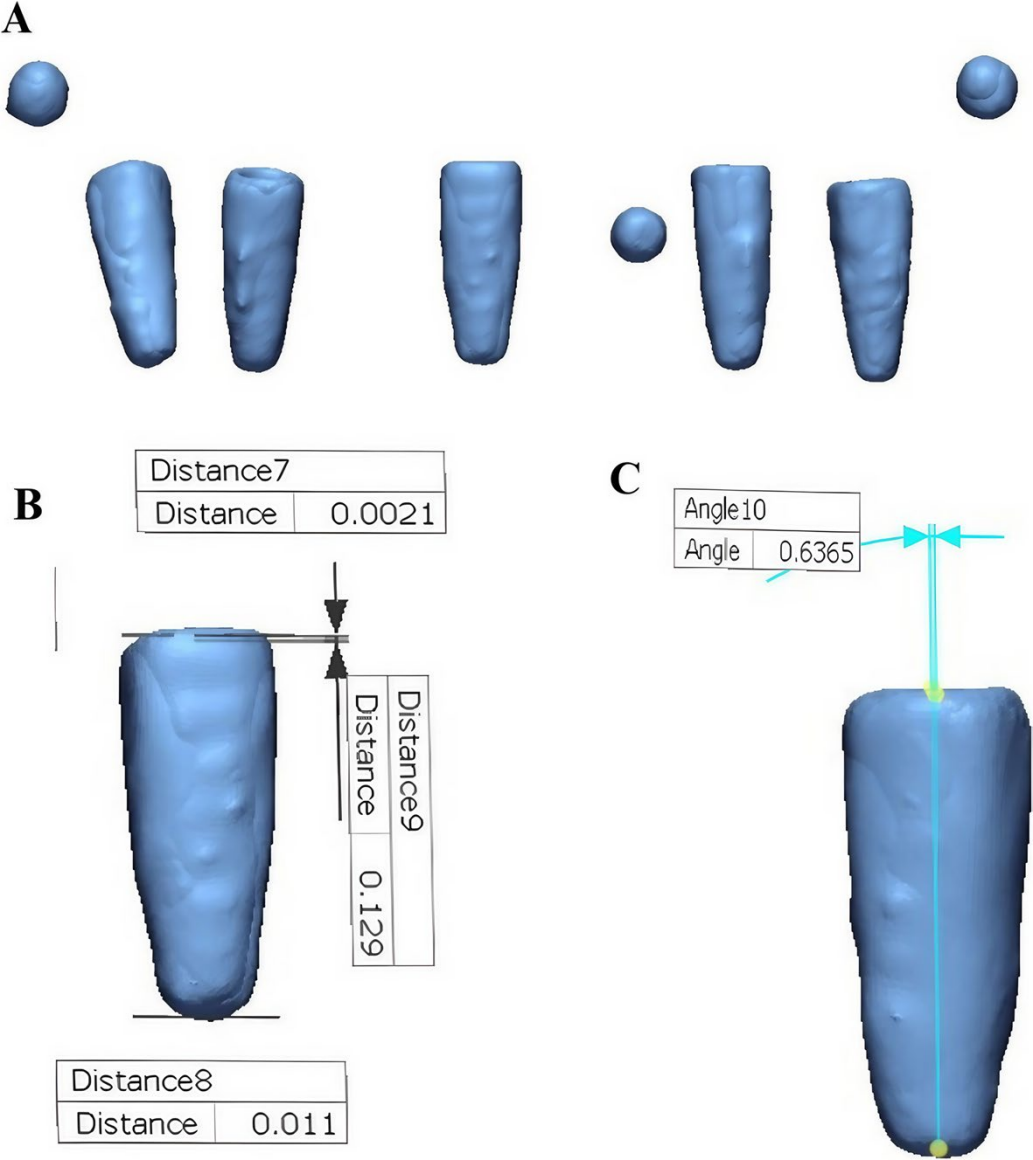
(**A**) Alignment of the reference image and the sample image, followed by measurement of (**B**) linear and (**C**) angular implant deviations

This process minimized registration error by relying on the complete model geometry, compensating for minor manual variations in beads placement. The aligned implants-and-beads STL files (Fig. 8) were then exported from ExoCAD and imported into reverse engineering software (Geomagic Control X, 3D Systems, Rock Hill, SC, USA) for quantitative measurement of implant positional deviations (Fig. 9). This alignment approach corrected small misalignments by utilizing stable anatomical features, enhancing accuracy and reducing distortion bias.

**Fig. 10:**
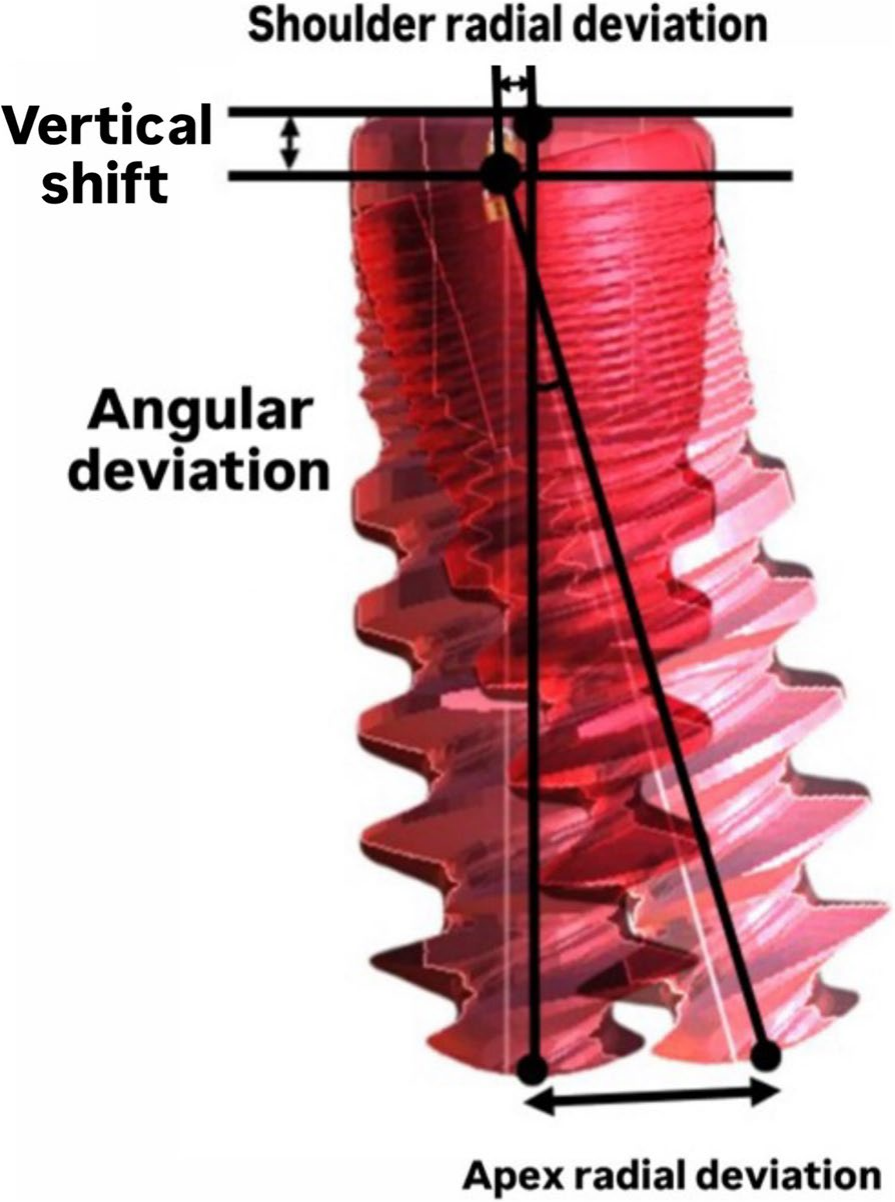
Visual illustration of the measured deviation parameters

Implant position accuracy was evaluated by assessing radial deviation, angular deviation, and vertical shift, with all values reported in microns. A visual representation of these measurements is provided (Fig. 10). Radial deviation, defined as the lateral displacement of the implant axis perpendicular to that of the reference model, was evaluated at two locations: the implant shoulder (shoulder radial deviation) and the implant apex (apex radial deviation). Angular deviation referred to the angle between the implant axes of the reference model and the poured casts. Vertical shift denoted the difference in height of the implant platform between the reference model and the casts.

### Statistical Analysis

Statistical analyses were performed using IBM SPSS Statistics version 27.0 (Armonk, NY, USA). Descriptive statistical measures, including mean, median, standard deviation, skewness, kurtosis, and confidence intervals, were calculated to summarize the data and assess distribution characteristics. To address potential distortions due to outliers, Huber’s M-estimator was employed as a robust measure of central tendency. Data distribution was further evaluated through visual inspection using histograms and quantile–quantile (Q-Q) plots.

Inferential analysis, One-Way ANOVA was used to assess differences among the three impression coping groups (mount, open-tray, and clips), with a significance threshold set at *p* < 0.05. When significant group differences were identified, post hoc comparisons were performed. Tukey’s HSD test was applied under the assumption of homogeneity of variances, while the Games-Howell test was used when this assumption was violated. Effect sizes were calculated using eta-squared (η^2^) to evaluate the practical significance of group differences.

## Results

The results showed a statistically significant difference in angular deviation (*p* < 0.001), apical deviation (*p* = 0.003), and vertical shift (*p* < 0.001) for Implant 1 (25°), and a statistically significant difference (*p* = 0.011) in vertical shift for Implant 2 (15°). In contrast, no significant differences were observed among the three impression coping techniques for any of the four parameters (coronal, apical, angular deviations, and vertical shift) at Implants 3, 4, and 5 (0° or straight implants). These findings are detailed in Table 1.

**Table 1:** One-way ANOVA of Deviation Measures across The Three Groups of Impression Copings (in μm)

Measure Implant	One-way ANOVA					
	Mount	Open Tray	Clips	*F*(*df1*,*df2*)	*p*	*η*^2^
**Angle Deviation**						
1	0.67 ± 0.178	0.32 ± 0.105	0.36 ± 0.098	20.180(2,27)^a^	< 0.001^**^	0.599
2	0.34 ± 0.104	0.25 ± 0.077	0.30 ± 0.057	3.099(2,27)^a^	0.061	0.187
3	0.34 ± 0.179	0.21 ± 0.063	0.22 ± 0.086	2.039(2,16.313)^b^	0.162	0.199
4	0.29 ± 0.149	0.19 ± 0.073	0.19 ± 0.120	1.748(2,16.429)^b^	0.205	0.140
5	0.29 ± 0.188	0.16 ± 0.091	0.23 ± 0.116	2.230(2,27)^a^	0.127	0.142
**Shoulder Deviation**						
1	43.10 ± 5.525	38.70 ± 6.350	38.89 ± 3.823	2.177(2,27)^a^	0.133	0.279
2	31.72 ± 9.321	31.70 ± 8.768	25.54 ± 10.532	0.971(2,27)^a^	0.391	0.065
3	25.26 ± 11.217	20.64 ± 11.859	16.24 ± 7.100	1.927(2,27)^a^	0.165	0.073
4	14.54 ± 8.234	18.58 ± 9.301	18.31 ± 10.663	0.570(2,27)^a^	0.572	0.038
5	21.69 ± 12.290	17.77 ± 10.019	13.71 ± 3.274	2.415(2,13.851)^b^	0.126	0.039
**Apical Deviation**						
1	51.85 ± 15.362	29.99 ± 16.902	29.28 ± 12.545	7.270(2,27)^a^	0.003^**^	0.350
2	32.49 ± 10.470	26.27 ± 6.644	28.52 ± 7.108	1.454(2,27)^a^	0.251	0.097
3	24.19 ± 18.839	16.33 ± 5.361	18.56 ± 8.233	0.912(2,15.751)^b^	0.422	0.075
4	32.85 ± 21.903	18.63 ± 8.832	22.81 ± 10.656	1.870(2,16.690)^b^	0.185	0.150
5	24.41 ± 17.088	23.13 ± 9.839	19.66 ± 6.440	0.624(2,16.129)^b^	0.548	0.030
**Vertical Shift**						
1	58.97 ± 14.903	37.50 ± 15.044	34.02 ± 7.986	10.692(2,27)^a^	< 0.001^**^	0.442
2	34.23 ± 5.783	25.63 ± 7.255	31.54 ± 4.601	5.417(2,27)^a^	0.011^*^	0.286
3	27.60 ± 13.222	20.25 ± 15.491	27.23 ± 13.713	0.853(2,27)^a^	0.437	0.059
4	31.04 ± 20.904	20.81 ± 16.605	19.44 ± 14.054	1.324(2,27)^a^	0.283	0.089
5	27.82 ± 18.507	15.70 ± 7.639	21.77 ± 10.652	2.308(2,16.486)	0.131	0.137

*Significant at 0.05

**Significant at 0.01

^a^F statistic from ANOVA Table.

^b^W statistic (Welch) from Robust Tests of Equality of Means Table

**Fig. 11:**
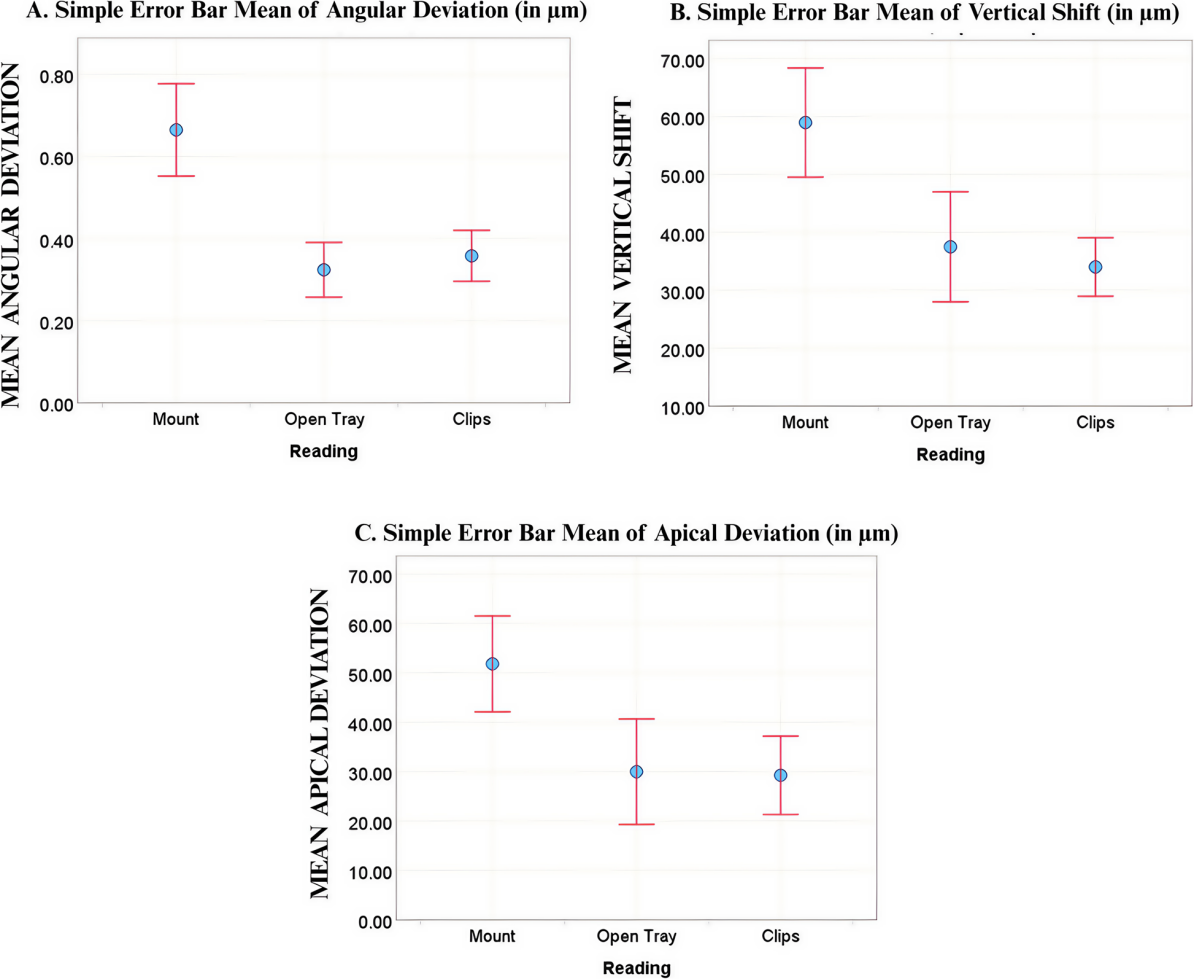
(**A**)Angular Deviation, (**B**) Vertical Shift, and (**C**) Apical Deviation Values at Implant 1 (25°)

**Fig. 12:**
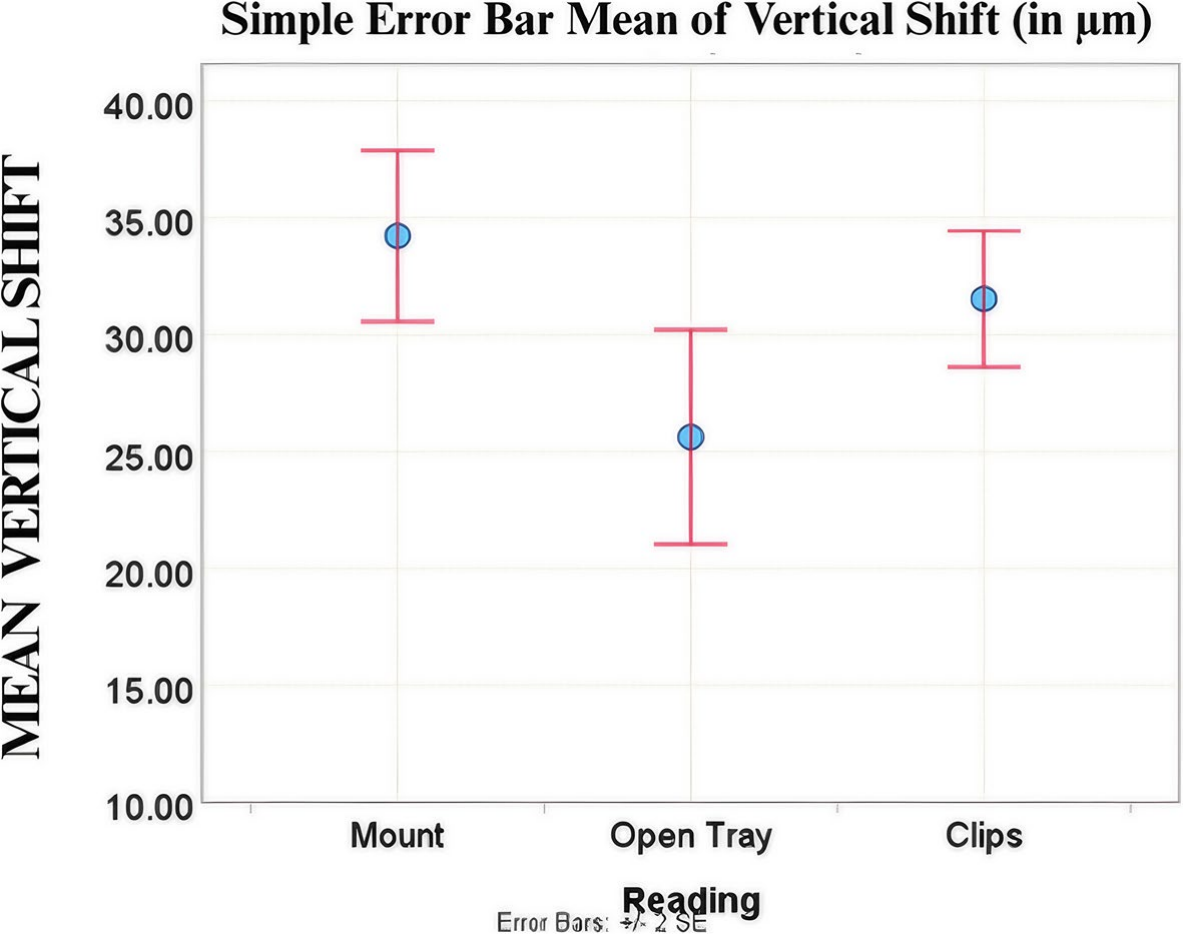
Vertical Shift Values in Vertical Group at Implant 2 (15°)

Upon pairwise comparison at Implant 1, the hexed implant mount exhibited higher angular deviation (0.67° ± 0.178°), apical deviation (51.85 ± 15.362 μm), and vertical shift (58.97 ± 14.903 μm) values compared to both the open-tray (0.32° ± 0.105°), (29.99μm ± 16.902 μm), and (37.50μm ± 15.044μm); and clips impression copings (0.36° ± 0.098°), (29.28μm ± 12.545μm), and (34.02μm ± 7.986 μm), respectively (Fig. 11). However, no significant difference was found between the open-tray and clips impression copings for any parameter. At Implant 2, a greater vertical shift was observed in the hexed implant mount group (34.23 ± 5.783μm) compared to the open-tray impression coping (25.63 ± 7.255μm) (Fig. 12). No significant differences were found in apical, coronal, or angular deviations between the hexed implant mount and the open-tray coping. Likewise, no differences were observed between the clips and open-tray copings for any of the parameters. These findings are detailed in Table 2.

**Table 2:** Post Hoc Multiple Comparisons of Deviation Measures across The Three Groups of Impression Copings

Implant	Reading Pair	Angle		Shoulder		Apical		Vertical Shift	
		MD	Sig	MD	Sig	MD	Sig	MD	Sig
**1**	**Mount < – > Open Tray**	**0.34**	< 0.001^**^	4.41	0.174	**21.85**	0.008^**^	**21.46**	0.003^**^
	**Mount < – > Clips**	**0.31**	< 0.001^**^	4.21	0.201	**22.57**	0.006^**^	**24.95**	< 0.001^**^
	**Clips < – > Open Tray**	0.03	0.835	0.20	0.996	−0.71	0.994	−3.49	0.823
**2**	**Mount < – > Open Tray**	**0.09**	0.049^*^	0.02	1.000	6.22	0.230	**8.60**	0.009^**^
	**Mount < – > Clips**	0.04	0.461	5.18	0.458	3.97	0.538	2.69	0.579
	**Clips < – > Open Tray**	0.05	0.415	−5.16	0.461	2.25	0.816	5.91	0.087
**3**	**Mount < – > Open Tray**	0.12	0.149	4.62	0.579	7.87	0.441	7.35	0.487
	**Mount < – > Clips**	0.12	0.178	9.02	0.141	5.63	0.671	0.36	0.998
	**Clips < – > Open Tray**	0.00	0.996	−4.40	0.610	2.24	0.756	6.98	0.521
**4**	**Mount < – > Open Tray**	0.10	0.183	−4.03	0.611	14.22	0.180	10.23	0.400
	**Mount < – > Clips**	0.09	0.300	−3.77	0.650	10.04	0.418	11.60	0.312
	**Clips < – > Open Tray**	0.01	0.990	−0.26	0.998	4.18	0.614	−1.37	0.983
**5**	**Mount < – > Open Tray**	0.13	0.107	3.92	0.719	1.29	0.977	12.12	0.177
	**Mount < – > Clips**	0.06	0.630	7.98	0.165	4.75	0.697	6.05	0.652
	**Clips < – > Open Tray**	0.07	0.473	−4.06	0.468	−3.47	0.629	6.07	0.333

*. MD is significant at the 0.05 level

**. MD is significant at the 0.01 level

## Discussion

Accurate implant impressions are crucial for transferring implant positions to the definitive cast, ensuring the passivity of implant-supported prostheses [1, 2]. This passivity ensures restoration longevity and maintains peri-implant tissue health. Impression accuracy is influenced by factors such as the type and material of the coping, splinting, implant angulation, and the number of implants [5–7, 17]. The study compared the accuracy of open-tray, clips, and hexed implant mounts in transferring implant positions for both straight and angulated implants. Statistically significant differences were found across all measured parameters (shoulder and apical radial deviation, angular deviation, and vertical shift); consequently, the null hypothesis has been rejected.

These findings highlight that impression coping type significantly influences implant position transfer accuracy, potentially affecting the passive fit and long-term clinical success of the prosthesis. The varying accuracy among open-tray, snap-on clips, and hexed implant mounts highlights the importance of selecting an impression technique suitable to specific clinical situations.

To assess the impact of implant angulation on impression accuracy, five implants were positioned in a mandibular epoxy resin model to replicate clinical conditions and stabilize the dental implants. The epoxy resin was chosen for its superior mechanical properties, dimensional stability, and a modulus of elasticity that closely resembles that of human bone [34, 35].

Accurate implant placement at the preplanned angulations was achieved using a surgical guide. Surgical guides have been shown to significantly enhance the precision of implant positioning [36–38]. Furthermore, recent studies have reported that sleeveless surgical guides, as utilized in the present study, demonstrate comparable accuracy to conventional guides equipped with metal sleeves [39, 40]. Recent advancements in digitally guided workflows have further optimized impression accuracy and prosthesis fit. For instance, the use of digital implant planning and transfer techniques has been shown to improve clinical outcomes and workflow efficiency [20, 27].

On the left side of the model, three straight implants were placed, including one at the midline, to assess whether its proximity to an adjacent angulated implant would influence its transfer accuracy. On the right side, two angulated implants were placed at 25° (15° distal and 10° buccal) and 15° mesial to create maximal divergence and assess the impact of high angulation on impression accuracy. A major concern in implant prosthodontics is achieving accurate impressions for angulated implants. Due to factors such as bone resorption, poor bone quality, hard tissue undercuts, and esthetics, achieving parallel implant placement can be challenging. In such cases, implants are often placed at buccolingual or mesiodistal angles. Impressions of angulated implants may be less accurate than those of parallel implants [6, 7, 17]. Previous studies, including those by Tsagkalidis et al. [41] and Tafti et al. [42], have examined the effect of 0°, 15°, and 25° implant angulation on impression accuracy, as these angles are frequently encountered in clinical practice.

In the present study, open-tray impression copings were used for their superior transfer accuracy [13, 43, 44]. However, they can be time-consuming and uncomfortable for both the patient and clinician, especially in cases with limited interarch space. To address this, clips impression copings and hexed implant mounts, used in the study, are classified as pick-up closed-tray implant-level impression copings. These copings remain within the impression, eliminating the need for unscrewing and repositioning of the copings, which could otherwise introduce positional inaccuracies. They combine the advantages of open-tray copings (direct), such as preventing repositioning errors, with those of closed-tray copings (indirect), including ease of use, time efficiency, improved patient comfort, and better accessibility in limited interarch spaces [1, 28, 45].

Clips impression copings connect to the abutment-implant interface with a frictional fit, and their short connection helps prevent displacement, particularly in angulated implants. A hexed implant mount, which also connects by frictional fit, differs from the clips impression coping in having a longer connection. While most previous research has focused on open-tray (direct) and closed-tray (indirect) transfer impression copings, pick-up closed-tray copings, as utilized in this study, have been insufficiently addressed.

The impression copings and implant mounts utilized in this study were made of titanium, which provide greater accuracy than plastic copings. Walker et al. [30] found that the metal copings technique yielded more accurate casts than the plastic caps technique. Additionally, Arieli et al. [46] concluded that plastic snap-on copings were less accurate due to dimensional distortions caused by acrylic resin shrinkage during splinting—a problem that persisted even after sectioning and rejoining the caps. The resilience of plastic components in closed-tray snap-on techniques may also be contributed to deformation from polymerization shrinkage [46].

Splinting was performed using low-shrinkage autopolymerizing resin to minimize micromovements of the copings during impression registration of multiple and angulated implants. The splinted copings were sectioned and reconnected to counteract polymerization shrinkage. Sectioning was performed 24 h after splinting to allow for most of the polymerization shrinkage to occur. Previous studies indicate that approximately 80% of shrinkage happens within the first 17 min, with total volumetric shrinkage reaching about 7.9% over 24 h. [31–33] Adell et al. [47] emphasized the significance of intraorally splinting impression copings before impression making. Similarly, Vigolo et al. [48] reported that definitive casts exhibited greater accuracy when impression copings were splinted using autopolymerizing resin. To further minimize polymerization shrinkage, it has been recommended to section and rejoin the autopolymerizing resin splint [49, 50].

VPES medium-viscosity, monophasic precision impression material (Identium Medium, Kettenbach SNC, France) was used to enhance accuracy. This material offers improved elasticity, making it particularly suitable for angulated implants, while its increased stiffness prevents tearing during impression removal. A systematic review by Saini et al. [51] found that VPES has significantly higher tensile strength and fewer defects compared to polyether (PE) and polyvinyl siloxane (PVS). VPES also demonstrated superior wettability and a lower contact angle with water, making it especially effective in moist clinical environments [52]. Singer et al. [53] and Rose et al. [54] further supported these findings, noting that VPES produced markedly better dimensional accuracy than both PE and PVS.

In the present study, a UTM was employed to control and standardize both the direction and force of tray removal, which was performed along a vertical axis. Terry et al. [55] introduced posterior lateral detachment wings on custom trays to achieve uniform axial traction during removal, while Geramipanah et al. [56] used extensions connected to a vertical surveyor rod to ensure controlled vertical detachment. In line with previous studies evaluating the tensile strength of impression materials, the crosshead speed of the universal testing machine was set at 5 mm/min [52, 57].

Implant position accuracy was evaluated by obtaining CBCT images of both the reference model and the poured casts. These images were superimposed in order to assess angular deviation, shoulder and apex radial deviation, and vertical shift. This method has been widely adopted in previous studies, as described by Li et al. [58] and Yi et al. [59], in which CBCT images were converted into STL files and analyzed using reverse engineering software such as Geomagic Control X (3D Systems, Rock Hill, SC, USA). Similarly, D’haese et al. [60] used the same software to compare the accuracy of conventional and digital impressions.

Open-tray impression copings demonstrated superior accuracy compared to hexed implant mounts in transferring the position of implant 1 (25°), particularly in terms of angular deviation, apical radial deviation, and vertical shift. For implant 2 (15°), they also showed reduced vertical shift. The linear and angular deviations associated with the hexed implant mount at 25° exceeded the clinically acceptable thresholds of 50 µm [17] and 0.4° [18, 19], respectively, as reported in previous studies, indicating reduced accuracy for highly angulated implants. However, the deviations observed with the hexed implant mount at 15° remained within these clinically acceptable limits [17–19].

These findings are consistent with the results of Tsagkalidis et al. [41], who reported that splinted open-tray impression techniques produced the highest accuracy for implants angled at 25°. However, unlike the present study, which utilized titanium copings, their comparison involved snap-on plastic caps, which may have reduced the accuracy. Similarly, Papaspyridakos et al. [13] found open-tray techniques to be more accurate in fully edentulous cases involving multiple implants with angulations exceeding 20°. Additional studies by Patil et al. [44] and Elshenawy et al. [43] also concluded that splinted direct (open-tray) techniques offer superior accuracy; however, their comparisons were against closed-tray transfer copings, which may limit the direct applicability of their results to the current findings.

Moreover, the use of the clips impression coping demonstrated greater accuracy for Implant 1 (25°) than the hexed implant mount, which may be attributed to the deeper connection of the hexed implant mount. A deeper connection could lead to displacement during impression removal, potentially causing distortion [61]. Sorrentino et al. [6] reported that for nonparallel implants, shorter connections yielded improved accuracy. However, the conclusions of Richi et al. [62] did not align with the technique used in our study, as they found that for angulated implant positions, the impressions with the superior accuracy were achieved using the non-hex splinted coping technique. They suggested that the 2 mm shorter connection area of the non-hex copings could reduce removal stress associated with implant divergence by minimizing the contact surface between the implant and coping. According to their explanation, this design potentially limits coping movement during tray removal, thereby enhancing impression accuracy [62].

In contrast, both open-tray and clips impression copings exhibited comparable accuracy for straight and angulated implants (25° and 15°), with all measured deviations remaining within clinically acceptable limits [17–19]. Baig et al. [63] reported conflicting evidence regarding the superiority of open-tray versus closed-tray techniques, with closed-tray technique utilizing transfer (indirect) copings. Lee et al. [8] suggested that while there is no significant difference between open- and closed-tray techniques for three or fewer implants, the transfer (indirect) technique improves accuracy when four or more implants are used, with the closed-tray technique in their study also utilizing transfer (indirect) copings. In contrast, the findings of the present study disagree with those of Tsagkalidis et al. [41], who found the snap-fit coping to be the least accurate for implant angulations between 0° and 25°. This discrepancy may stem from dimensional distortion of the plastic caps caused by acrylic resin shrinkage and the deformation caused by the flexibility of plastic components during polymerization [46].

In addition, no significant difference in transfer accuracy was observed between the hexed implant mount and the other copings for straight implants. Notably, the straight implant positioned at the midline was not affected by its proximity to the adjacent angulated implant. This finding is consistent with previous studies [46, 64–66], which reported that closed-tray snap-on techniques using plastic snap caps achieved accuracy comparable to that of open-tray techniques for straight implants. Similarly, Rashidan et al. [61] found no significant difference between open-tray and closed-tray techniques in such cases, further supporting our observation. This outcome may be attributed to the fact that tray removal in the case of straight implants does not introduce significant stresses, thereby minimizing distortion and resulting in comparable accuracy across different coping types.

Vertical tray removal simulated removal from the flanges, which was perpendicular to the occlusal plane, with removal of the straight copings followed a parallel path to their direction of removal. In the study by Tafti et al. [42] and Balamurugan et al. [67], the direction of removal was not standardized, which may explain their finding that the snap-on technique was less accurate than the open-tray technique in terms of linear and angular displacements. This discrepancy could be attributed to the direction of tray removal.

The findings of this study have important clinical implications. Understanding the influence of impression coping design on the accuracy of implant position transfer can guide clinicians in selecting the most suitable technique based on specific clinical scenarios, particularly in cases involving angulated implants or limited interarch space. The demonstrated superiority of open-tray and clips copings for angulated implants supports their use in situations where implant divergence is unavoidable. Additionally, the comparable accuracy between open-tray and pick-up closed-tray copings for straight implants suggests that clinicians may choose either option based on patient comfort and access.

However, it is important to acknowledge that the results of this in vitro study may not fully replicate outcomes in a clinical environment. In vivo conditions introduce additional variables such as patient movement, soft tissue interference, limited visibility, saliva contamination, and variations in temperature and humidity—all of which can affect the handling of impression materials and overall accuracy. Moreover, anatomical constraints and operator variability may influence the performance of impression techniques. A further limitation of the present methodology lies in the manual positioning of the metal reference beads, which, despite careful execution and verification, may introduce minor inconsistencies that could affect alignment precision. Therefore, while the current study provides valuable insights under controlled conditions, further clinical research is necessary to validate these findings in real-world settings and to assess their direct impact on prosthesis fit and long-term success.

## Conclusion

Based on the findings of this in vitro study, the following conclusions can be drawn:

1. Open-tray and clips impression copings demonstrated significant efficiency as implant-level impression copings for both straight and angulated implants up to 25°, without any detrimental effect on transfer accuracy.
2. The hexed implant mount proved to be a viable implant-level impression coping for straight implants and those of 15° angulations, associated with minimal deviations (displacement < 50 µm; angular deviation < 0.4°), indicating acceptable accuracy.
3. Vertical tray removal is recommended, as it consistently resulted in reduced implant deviations and enhanced impression accuracy. This method minimizes the stress on the impression copings, reducing displacement and improving the overall transfer of implant positions, particularly for angulated implants.

## Data Availability

The datasets used and/or analyzed during the current study are available from the corresponding author upon reasonable request.

## Abbreviations

CAD-CAM: Computer-aided design and computer-aided manufacturing
3D: 3-Dimensional
DICOM: Digital Imaging and Communications in Medicine
STL: Standard Tessellation Language
UTM: Universal testing machine
VPES: Vinyl polyether siloxane
CBCT: Cone beam computed tomography
Tukey’s HSD: Tukey’s honestly significant difference
ANOVA: Analysis of variance
MD: Mean Difference

## References

[1] Carr AB. Comparison of impression techniques for a five-implant mandibular model. Int J Oral Maxillofac Implants. 1991;6(4):448–55. 1820314

[2] Wee AG, Aquilino SA, Schneider RL. Strategies to achieve fit in implant prosthodontics: a review of the literature. Int J Prosthodont. 1999;12(2):167–78. 10371920

[3] Del’Acqua MA, Chávez AM, Amaral ÂLC, Compagnoni MA. Comparison of impression techniques and materials for an implant-supported prosthesis. J Prosthet Dent. 2010;103(6):378–83. 20657873

[4] Papaspyridakos P, Lal K, White GS, Weber HP, Gallucci GO. Effect of splinted and nonsplinted impression techniques on the accuracy of fit of fixed implant prostheses in edentulous patients: a comparative study. Int J Oral Maxillofac Implants. 2011;26(6):1267–72. 22167432

[5] Conrad HJ, Pesun IJ, DeLong R, Hodges JS. Accuracy of two impression techniques with angulated implants. J Prosthet Dent. 2007;97(6):349–56. 17618917 10.1016/S0022-3913(07)60023-7

[6] Sorrentino R, Gherlone EF, Calesini G, Zarone F. Effect of implant angulation, connection length, and impression material on the dimensional accuracy of implant impressions: an in vitro comparative study. Clin Implant Dent Relat Res. 2010;12(Suppl 1):e63–76. 19438937 10.1111/j.1708-8208.2009.00167.x

[7] Mpikos P, Kafantaris N, Tortopidis D, Galanis C, Kaisarlis G, Koidis P. The effect of impression technique and implant angulation on the impression accuracy of external- and internal-connection implants. Int J Oral Maxillofac Implants. 2012;27(6):1422–8. 23189292

[8] Lee H, So JS, Hochstedler JL, Ercoli C. The accuracy of implant impressions: a systematic review. J Prosthet Dent. 2008;100(4):285–91. 18922257 10.1016/S0022-3913(08)60208-5

[9] Suphangul S, Pujarern P, Rokaya D, Kanchanasobhana C, Rungsiyakull P, Chaijareenont P. Comparison of plaque accumulation between titanium and PEEK healing abutments. J Funct Biomater. 2024;15(11):334. 39590538 10.3390/jfb15110334 PMC11595035

[10] Moreira AH, Rodrigues NF, Pinho AC, Fonseca JC, Vilaça JL. Accuracy comparison of implant impression techniques: a systematic review. Clin Implant Dent Relat Res. 2015;17(Suppl 2):e751–64. 25828851 10.1111/cid.12310

[11] Arora A, Upadhyaya V, Parashar KR, Malik D. Evaluation of the effect of implant angulations and impression techniques on implant cast accuracy: an in vitro study. J Indian Prosthodont Soc. 2019;19(2):149–58. 31040549 10.4103/jips.jips_337_18 PMC6482616

[12] Osman MS, Ziada HM, Abubakr NH, Suliman AM. Implant impression accuracy of parallel and non-parallel implants: a comparative in-vitro analysis of open and closed tray techniques. Int J Implant Dent. 2019;5(1):4. 30778790 10.1186/s40729-019-0159-5 PMC6379502

[13] Papaspyridakos P, Chen CJ, Gallucci GO, Doukoudakis A, Weber HP, Chronopoulos V. Accuracy of implant impressions for partially and completely edentulous patients: a systematic review. Int J Oral Maxillofac Implants. 2014;29(4):836–45. 25032763 10.11607/jomi.3625

[14] Assif D, Marshak B, Schmidt A. Accuracy of implant impression techniques. Int J Oral Maxillofac Implants. 1996;11(2):216–22. 8666454

[15] Naert I, Quirynen M, van Steenberghe D, Darius P. A study of 589 consecutive implants supporting complete fixed prostheses. Part II: Prosthetic aspects. J Prosthet Dent. 1992;68(6):949–56. 1494126 10.1016/0022-3913(92)90557-q

[16] Carlsson B, Carlsson GE. Prosthodontic complications in osseointegrated dental implant treatment. Int J Oral Maxillofac Implants. 1994;9(1):90–4. 8150518

[17] Assunção WG, Filho HG, Zaniquelli O. Evaluation of transfer impressions for osseointegrated implants at various angulations. Implant Dent. 2004;13(4):358–66. 15591998 10.1097/01.id.0000144509.58901.f7

[18] Knechtle N, Wiedemeier D, Mehl A, Ender A. Accuracy of digital complete-arch, multi-implant scans made in the edentulous jaw with gingival movement simulation: an in vitro study. J Prosthet Dent. 2022;128(3):468–78. 33612335 10.1016/j.prosdent.2020.12.037

[19] Huang R, Liu Y, Huang B, Zhang C, Chen Z, Li Z. Improved scanning accuracy with newly designed scan bodies: an in vitro study comparing digital versus conventional impression techniques for complete-arch implant rehabilitation. Clin Oral Implants Res. 2020;31(7):625–33. 32181919 10.1111/clr.13598

[20] Talesara V, Bennani V, Aarts J, Ratnayake J, Khurshid Z, Brunton P. Accuracy of digitally coded healing abutments: a systematic review. Saudi Dent J. 2023;35(8):891–903. 38107040 10.1016/j.sdentj.2023.08.006 PMC10724348

[21] Çakmak G, Yilmaz H, Treviño A, Kökat AM, Yilmaz B. The effect of scanner type and scan body position on the accuracy of complete-arch digital implant scans. Clin Implant Dent Relat Res. 2020;22(4):533–41. 32643259 10.1111/cid.12919

[22] Mangano FG, Veronesi G, Hauschild U, Mijiritsky E, Mangano C. Trueness and precision of four intraoral scanners in oral implantology: a comparative in vitro study. PLoS ONE. 2016;11(9): e0163107. 27684723 10.1371/journal.pone.0163107 PMC5042463

[23] Wulfman C, Naveau A, Rignon-Bret C. Digital scanning for complete-arch implant-supported restorations: A systematic review. J Prosthet Dent. 2020;124(2):161–7. 31757443 10.1016/j.prosdent.2019.06.014

[24] Di Fiore A, Meneghello R, Graiff L, Savio G, Vigolo P, Monaco C, et al. Full arch digital scanning systems performances for implant-supported fixed dental prostheses: a comparative study of 8 intraoral scanners. J Prosthodont Res. 2019;63(4):396–403. 31072730 10.1016/j.jpor.2019.04.002

[25] Mizumoto RM, Yilmaz B. Intraoral scan bodies in implant dentistry: A systematic review. J Prosthet Dent. 2018;120(3):343–52. 29627211 10.1016/j.prosdent.2017.10.029

[26] Keul C, Güth J-F. Accuracy of full-arch digital impressions: an in vitro and in vivo comparison. Clin Oral Investig. 2020;24(2):735–45. 31134345 10.1007/s00784-019-02965-2

[27] Ali K, Alzaid AA, Suprono MS, Garbacea A, Savignano R, Kattadiyil MT. Evaluating the effects of splinting implant scan bodies intraorally on the trueness of complete arch digital scans: A clinical study. J Prosthet Dent. 2024;132(4):781.e1–e7. 38594088 10.1016/j.prosdent.2024.03.004

[28] Carr AB. Comparison of impression techniques for a two-implant 15-degree divergent model. Int J Oral Maxillofac Implants. 1992;7(4):468–75. 1299642

[29] Nissan J, Ghelfan O. The press-fit implant impression coping technique. J Prosthet Dent. 2009;101(6):413–4. 19463669 10.1016/S0022-3913(09)60088-3

[30] Walker MP, Ries D, Borello B. Implant cast accuracy as a function of impression techniques and impression material viscosity. Int J Oral Maxillofac Implants. 2008;23(4):669–74. 18807563

[31] Jain S, Sayed ME, Khawaji RAA, Hakami GAJ, Solan EHM, Daish MA, et al. Accuracy of 3 intraoral scanners in recording impressions for full arch dental implant-supported prosthesis: an in vitro study. Med Sci Monit. 2024;30:e946624. 39645575 10.12659/MSM.946624 PMC11636004

[32] Giuliodori G, Rappelli G, Aquilanti L. Intraoral scans of full dental arches: An in vitro measurement study of the accuracy of different intraoral scanners. Int J Environ Res Public Health. 2023;20(6):4776. 36981684 10.3390/ijerph20064776 PMC10048864

[33] Huang L, Liu L, Yang S, Khadka P, Zhang S. Evaluation of the accuracy of implant placement by using implant positional guide versus freehand: a prospective clinical study. Int J Implant Dent. 2023;9(1):45. 38036932 10.1186/s40729-023-00512-z PMC10689697

[34] Afshari A, Shahmohammadi R, Mosaddad SA, Pesteei O, Hajmohammadi E, Rahbar M, et al. Free-hand versus surgical guide implant placement. Adv Mater Sci Eng. 2022;2022:6491134.

[35] Younis H, Lv C, Xu B, Zhou H, Du L, Liao L, et al. Accuracy of dynamic navigation compared to static surgical guides and the freehand approach in implant placement: a prospective clinical study. Head Face Med. 2024;20(1):30. 38745297 10.1186/s13005-024-00433-1 PMC11092008

[36] Thangwarawut P, Amornvit P, Rokaya D, Kiattavorncharoen S. Comparison of different types of static computer-guided implant surgery in varying bone inclinations. Materials. 2022;15(9):3004. 35591339 10.3390/ma15093004 PMC9103329

[37] Adams CR, Ammoun R, Deeb GR, Bencharit S. Influence of metal guide sleeves on the accuracy and precision of dental implant placement using guided implant surgery: An in vitro study. J Prosthodont. 2023;32(1):62–70. 35257456 10.1111/jopr.13503 PMC10078659

[38] Tsagkalidis G, Tortopidis D, Mpikos P, Kaisarlis G, Koidis P. Accuracy of 3 different impression techniques for internal connection angulated implants. J Prosthet Dent. 2015;114(4):517–23. 26213265 10.1016/j.prosdent.2015.05.005

[39] Tafti AF, Hatami M, Razavi F, Ebadian B. Comparison of the accuracy of open-tray and snap-on impression techniques of implants with different angulations. Dent Res J (Isfahan). 2019;16(6):413–20. 31803388 PMC6873238

[40] Elshenawy EA, Alam-Eldein AM, Abd Elfatah FA. Cast accuracy obtained from different impression techniques at different implant angulations (in vitro study). Int J Implant Dent. 2018;4(1):9. 29556841 10.1186/s40729-018-0118-6 PMC5859005

[41] Patil R, Kadam P, Oswal C, Patil S, Jajoo S, Gachake A. A comparative analysis of the accuracy of implant master casts fabricated from two different transfer impression techniques. J Int Soc Prev Community Dent. 2016;6(2):142–8. 27114954 10.4103/2231-0762.178747 PMC4820574

[42] Ebadian B, Rismanchian M, Dastgheib B, Bajoghli F. Effect of different impression materials and techniques on the dimensional accuracy of implant definitive casts. Dent Res J (Isfahan). 2015;12(2):136–43. 25878678 PMC4387625

[43] Arieli A, Adawi M, Masri M, Weinberg E, Beitlitum I, Pilo R, et al. The accuracy of open-tray vs. snap on impression techniques in a 6-implant model: An in vitro 3D study. Materials. 2022;15(6):2103. 35329555 10.3390/ma15062103 PMC8950925

[44] Mojon P, Oberholzer J-P, Meyer J-M, Belser UC. Polymerization shrinkage of index and pattern acrylic resins. J Prosthet Dent. 1990;64(6):684–8. 2079675 10.1016/0022-3913(90)90296-o

[45] Vigolo P, Fonzi F, Majzoub Z, Cordioli G. An evaluation of impression techniques for multiple internal connection implant prostheses. J Prosthet Dent. 2004;92(5):470–6. 15523336 10.1016/j.prosdent.2004.08.015

[46] Assif D, Fenton A, Zarb G, Schmitt A. Comparative accuracy of implant impression procedures. Int J Periodontics Restorative Dent. 1992;12(2):112–21. 1521993

[47] Adell R, Lekholm U, Rockler B, Brånemark PI. A 15-year study of osseointegrated implants in the treatment of the edentulous jaw. Int J Oral Surg. 1981;10(6):387–416. 6809663 10.1016/s0300-9785(81)80077-4

[48] Vigolo P, Majzoub Z, Cordioli G. Evaluation of the accuracy of three techniques used for multiple implant abutment impressions. J Prosthet Dent. 2003;89(2):186–92. 12616240 10.1067/mpr.2003.15

[49] Tarib N, Seong T, Chuen K, Kun M, Ahmad M, Kamarudin K. Evaluation of splinting implant impression techniques: two dimensional analyses. Eur J Prosthodont Restor Dent. 2012;20(1):35. 22474935

[50] Yamamoto E, Marotti J, de Campos TT, Neto PT. Accuracy of four transfer impression techniques for dental implants: a scanning electron microscopic analysis. Int J Oral Maxillofac Implants. 2010;25(6):1115–24. 21197487

[51] Saini RS, Alshadidi AAF, Hassan SAB, Aldosari LIN, Mosaddad SA, Heboyan A. Properties of a novel composite elastomeric polymer vinyl polyether siloxane in comparison to its parent materials: a systematic review and meta-analysis. BMC Oral Health. 2024;24(1):54. 38195442 10.1186/s12903-023-03830-1 PMC10775522

[52] Koseoglu M, Tugut F, Akin H. Tensile bond strength of soft and hard relining materials to conventional and additively manufactured denture-base materials. J Prosthodont. 2023;32(S1):74–80. 36111532 10.1111/jopr.13608

[53] Singer L, Habib SI, Shalaby HEA, Saniour SH, Bourauel C. Digital assessment of properties of the three different generations of dental elastomeric impression materials. BMC Oral Health. 2022;22(1):379. 36064393 10.1186/s12903-022-02419-4 PMC9442984

[54] Rose S, Aravindakshan S, Mohamed Usman JA, Mohamed R, Menon S, Shafiullah RS, Salloum MG. Comparative evaluation of surface detail reproduction and dimensional stability of poly ether, vinyl siloxane, and vinyl siloxane ether impression materials: An in vitro study. J Pharm Bioallied Sci. 2021;13(Suppl 1):S851–6. 34447214 10.4103/jpbs.JPBS_819_20 PMC8375845

[55] Terry DA, Tric O, Blatz M, Burgess JO. The custom impression tray: fabrication and utilization. J Prosthet Dent. 2000;83(2):141–5. 20333863

[56] Geramipanah F, Sahebi M, Davari M, Hajimahmoudi M, Rakhshan V. Effects of impression levels and trays on the accuracy of impressions taken from angulated implants. Clin Oral Implants Res. 2015;26(9):1098–105. 24934081 10.1111/clr.12410

[57] Yi M-H, Shim J-S, Lee K-W, Chung M-K. Drying time of tray adhesive for adequate tensile bond strength between polyvinylsiloxane impression and tray resin material. J Adv Prosthodont. 2009;1(2):63–7. 21165257 10.4047/jap.2009.1.2.63 PMC2994680

[58] Li S, Yi C, Yu Z, Wu A, Zhang Y, Lin Y. Accuracy assessment of implant placement with versus without a CAD/CAM surgical guide by novices versus specialists via the digital registration method: an in vitro randomized crossover study. BMC Oral Health. 2023;23(1):426. 37370027 10.1186/s12903-023-03116-6 PMC10294323

[59] Yi C, Li S, Wen A, Wang Y, Zhao Y, Zhang Y. Digital versus radiographic accuracy evaluation of guided implant surgery: an in vitro study. BMC Oral Health. 2022;22(1):540. 36424579 10.1186/s12903-022-02585-5 PMC9694847

[60] D’Haese R, Vrombaut T, Roeykens H, Vandeweghe S. In vitro accuracy of digital and conventional impressions for full-arch implant-supported prostheses. J Clin Med. 2022;11(3):594. 35160045 10.3390/jcm11030594 PMC8836695

[61] Rashidan N, Alikhasi M, Samadizadeh S, Beyabanaki E, Kharazifard MJ. Accuracy of implant impressions with different impression coping types and shapes. Clin Implant Dent Relat Res. 2012;14(2):218–25. 19804420 10.1111/j.1708-8208.2009.00241.x

[62] Richi MW, Kurtulmus-Yilmaz S, Ozan O. Comparison of the accuracy of different impression procedures in case of multiple and angulated implants. Head Face Med. 2020;16(1):9. 32366261 10.1186/s13005-020-00225-3 PMC7197148

[63] Baig MR. Accuracy of impressions of multiple implants in the edentulous arch: a systematic review. Int J Oral Maxillofac Implants. 2014;29(4):869–80. 25032767 10.11607/jomi.3233

[64] Nakhaei M, Madani AS, Moraditalab A, Haghi HR. Three-dimensional accuracy of different impression techniques for dental implants. Dent Res J (Isfahan). 2015;12(5):431. 26604956 10.4103/1735-3327.166190 PMC4630706

[65] Akça K, Cehreli MC. Accuracy of 2 impression techniques for ITI implants. Int J Oral Maxillofac Implants. 2004;19(4):517–23. 15346748

[66] Özçelik T, Özcan I, Ozan O. Digital evaluation of the dimensional accuracy of four different implant impression techniques. Niger J Clin Pract. 2018;21(10):1247–53. 30297554 10.4103/njcp.njcp_284_17

[67] BalaMurugan T, Manimaran P. Evaluation of accuracy of direct transfer snap-on impression coping closed tray impression technique and direct transfer open tray impression technique: An in vitro study. J Indian Prosthodont Soc. 2013;13(3):226–32. 24431738 10.1007/s13191-012-0141-x PMC3732721

